# Inhibition of osteosarcoma by European Mistletoe derived val-miR218

**DOI:** 10.20517/evcna.2023.15

**Published:** 2023-07-03

**Authors:** Wenyan Xie, Catharina Delebinski, Dennis Gürgen, Maik Schröder, Georg Seifert, Matthias F. Melzig

**Affiliations:** ^1^Institute of Pharmacy, Freie Universität Berlin, Berlin 14195, Germany.; ^2^Department of Pediatric Oncology/Hematology, Otto-Heubner-Centre for Pediatric and Adolescent Medicine (OHC), Charité – Universitätsmedizin Berlin, corporate member of Freie Universität Berlin and Humboldt Universität zu Berlin, Berlin 13353, Germany.; ^3^EPO GmbH Berlin, Berlin 13353, Germany.; ^#^Authors contributed equally.

**Keywords:** miRNAs, medicinal plants, mistletoe, *Viscum album* L, osteosarcoma

## Abstract

**Aim:**

In recent years, there has been a growing interest in the therapeutic potential of plant-derived miRNAs, which have been considered new bioactive ingredients in medicinal plants. *Viscum album* L., commonly used as an adjuvant cancer therapy in central Europe, contains a large number of miRNAs associated with human diseases such as cancer, cardiovascular diseases, and neurological disorders. This study aimed to investigate whether mistletoe miRNAs, specifically val-miR218, exert anti-cancer activity against osteosarcoma.

**Methods:**

The anti-cancer effects of miRNAs from *V. album* L. were evaluated. The targets of val-miR218 were identified by RNA-seq. The mRNA and protein expression of the targets was confirmed by RT-qPCR and western blot analyses. The interaction between the val-miR218 and miRNA recognition elements (MREs) was validated by the dual-luciferase assay. The inhibitory effect of val-miR218 against osteosarcoma was investigated *in vivo*.

**Results:**

Among these abundant miRNAs in *V. album* L., val-miR218 showed high potential anti-cancer effects against osteosarcoma. To clarify its molecular mechanism of action, we sequenced val-miR218 associated RNAs and their down-regulated RNAs. As a result, 61 genes were considered the direct targets of val-miR218. Interestingly, these targets were related to essential cellular functions such as cell cycle, DNA replication, and cell morphology, suggesting that val-miR218 significantly inhibited cell growth and arrested osteosarcoma cells in G0/G1 phase by influencing basic cell activities. Mistletoe extracellular vesicles offered val-miR218 adequate protection and facilitated the uptake of val-miR281 by human cells. Moreover, val-miR218 showed significant anti-tumor effects *in vivo*.

**Conclusion:**

This study demonstrated the significant potential of val-miR218 regarding proliferation inhibition in various tumor cell lines *in vitro* and for osteosarcoma *in vivo*. Due to the increasing problems during chemotherapy, new therapeutic approaches are becoming more critical. The significant anti-cancer effects of medicinal plants derived miRNAs indicate a promising therapeutic strategy for treating cancer.

## INTRODUCTION

MicroRNAs (miRNAs) are naturally occurring, small single-stranded non-coding RNA molecules that regulate gene expression by binding to various parts of mRNA (5’UTR, CDS, and 3’UTR), inducing their cleavage or translational repression^[1,2]^. They are essential in various biological events, including cell proliferation, metabolism, development, and survival^[3,4]^. MiRNAs can be secreted and transferred into neighboring or distant cells via extracellular vesicles (EVs) or lipid-based carriers, thereby modulating recipient cell function^[5-7]^. Consequently, extracellular miRNAs act as messengers and effectors of intercellular communication involved in various physiological and pathological processes^[8,9]^. MiRNAs exist in multiple body fluids, and dysregulation of miRNA is associated with many human diseases, making miRNA promising diagnostic markers, and potential powerful candidates for therapeutic intervention^[10,11]^.

In both plants and animals, miRNA-mediated gene silencing is biochemically based on the principle of recognition and loading of miRNAs onto the argonaute (AGO) proteins to form an RNA-protein complex that targets complementary mRNAs and regulates protein synthesis^[1,2]^. Although the miRNAs of plants and animals appear to have evolved independently^[12]^, the AGOs are deeply conserved in many organisms^[13,14]^. The boundaries between plant and animal miRNAs in terms of mechanisms of miRNA-mediated action are becoming blurry, as there is increasing evidence showing the cross-kingdom regulation by miRNAs^[15-21]^. For example, miR168a derived from *Oryza sativa* L. was abundant in human serum or plant-feeding mammals. Bioinformatics analysis indicated its capacity to bind to the mRNA of low-density lipoprotein receptor adapter protein 1 (LDLRAP1) that controls blood cholesterol levels, which was confirmed *in vivo*^[15]^. Another study reported that miR2911 from honeysuckle (*Lonicera japonica* Thunb.) decoction directly targeted various influenza A viruses and protected mice from influenza virus infection^[16]^. Subsequent studies have shown that miR2911 inhibited SARS-CoV-2 replication and accelerated the negative conversion of infected patients^[19]^.

Furthermore, plant miR159, derived from either raw or cooked vegetables (e.g., broccoli, soybean), significantly suppressed breast cancer cell growth *in vitro* and *in vivo* by targeting transcription factor 7 (TCF7)^[17]^. Recently, Chen *et al.* identified 35 miRNAs that mapped to plants in human blood samples. They were able to show body fluid/tissue specificity and provided more evidence of the cross-kingdom regulatory function of plant miRNAs^[18]^. MiRNA171 is a widespread and conserved plant miRNA. Recent research has discovered that miRNA171 can decrease both the mRNA and protein levels of G protein subunit alpha 12 in human embryonic kidney 293 cells, which can then affect downstream signaling factors such as the mTOR pathway^[20]^. Li *et al.* indicated that miR167e-5p and miR156 could modulate the Wnt/β-catenin pathway and maintain intestinal epithelium homeostasis^[22,23]^. These results strengthen the beneficial and therapeutic effects against various pathological conditions.

*Viscum album* L. (*V. album*, Salantaceae), commonly known as mistletoe or European mistletoe, is a hemi-parasitic evergreen shrub that grows on many host trees, including apple, oak, pine, and other trees. Mistletoe preparations (i.e., abnobaVISCUM, Helixor, Iscador, Iscucin, and Isorel) are frequently used as adjuvant cancer therapy in central Europe^[24]^. Our previous studies revealed a large number of miRNAs presented in *V. album* L., including conserved and mistletoe-specific miRNAs. The bioinformatics analysis indicated that these miRNAs are associated with human diseases such as cancer, cardiovascular diseases, and neurological disorders^[25]^. Some mistletoe miRNAs were found stable during the herbal preparation process^[26]^, indicating their integrity prior to gastrointestinal digestion or subcutaneous injection, which are the common application routes for mistletoe preparations^[27,28]^. In this study, we screened eight miRNAs (three conserved miRNAs and five mistletoe-specific miRNAs) with relatively high expression in mistletoe for their anti-tumor effects, identified the most effective one, and explored potential human targets in order to investigate the possibility of mistletoe miRNAs as anti-cancer agents.

## METHODS

### Cell culture and miRNA mimics

The U2OS, SAOS-2, HepG2, and HT29 cell lines were obtained from the German Collection of Microorganisms and Cell Cultures GmbH (Leibniz Institute DMSZ, Braunschweig, Germany). The cell lines 143B, Colo320R, U-87, and SK-N-SH were purchased from American Type Culture Collection (ATCC, Manassas, VA, USA). Mesenchymal stem cells (MSCs) were obtained from Merck Millipore (Burlington, MA, USA). Cells were cultured with recommended media supplied with 10 % fetal bovine serum (FBS) in a humidified 5 % CO2 incubator at 37 °C. Human embryonic lung fibroblasts Fi301 (kindly offered by Prof. Dr. Christian Hagemeier, Labor für Pädiatrische Molekularbiologie, Charité Universitätsmedizin Berlin, Germany) were maintained in Eagle's minimum essential medium (EMEM) supplemented with Earle's balanced salt solution, 25 mM HEPES, 1 mM sodium pyruvate, 2 mM L-alanyl-L-glutamine, non-essential amino acids, 0.75 ‰ (w/v) sodium bicarbonate, 50 µg/mL gentamicin and 10 % fetal bovine serum (FBS).

The Allstar negative control miRNA (NC miRNA), biotinylated val-miR218, val-miR218 inhibitor and negative control inhibitor were designed and purchased from Qiagen (Hilden, Germany). The miRNA mimics [Table 1] were synthesized by Metabion International AG (Planegg/Steinkirchen, Germany).

**Table 1:** Sequence of miRNA mimics

**miRNA mimics**	**Sense (5' to 3' direction)**	**Anti-sense (5' to 3' direction)**
miR166a	UCGGACCAGGCUUCAUUCCC(2'OMe-C)	GGAAUGUUGUCUGGCUCGAG(2'OMe-G)
miR396a	UUCCACAGCUUUCUUGAACU(2'OMe-G)	GUUCAAUAAAGCUGUGGGAA(2'OMe-G)
miR159	UUUGGAUUGAAGGGAGCUCU(2’OMe-A)	GAGCUCCUUGAAGUCCAAUU(2’OMe-G)
val-miR218	GAUGAUCGCCACGUCGGAGG(2’OMe-A)	CUCCGACGUGGCGAUCAUCC(2’OMe-C)
val-miR11	CACUGUAGCACUUUUGACAAA(2'OMe-G)	UUGUCAAAAGUGCUACAGCGC(2'OMe-U)
val-miR1338	CGCAAGGACGUUAAUGAUGA(2'OMe-U)	CAUCAUUAACGUCCUUGAGA(2'OMe-C)
val-miR856	UAAUGGUGCUGGUUCAUGAUC(2'OMe-A)	AUCAUGCCAGUACCACUAC(2'OMe-C)
val-miR718	UUUUGUCUUUGUAGCAUGCU(2'OMe-U)	GUCUGCUCCAAAUACAAAAC(2'OMe-C)

### Transfection and viability assays

MiRNA transfection was performed by using Invitrogen™ Lipofectamin RNAiMAX Reagent (ThermoFisher Scientific, Waltham, MA, USA) according to the manufacturer’s instructions.

For cell viability assays, cells were seeded in 48-well plates at a concentration of 2 × 10^4^/well. After 24 h incubation, cells were transfected with miRNA mimics (20 nM). Cell viability was measured by using MTT assay at 72 h post-transfection.

For real-time cell proliferation analysis, cells were seeded at a density of 2 × 10^4^ cells per well in 8-well E-Plate (ACEA Biosciences; San Diego, CA, USA), and cell proliferation was monitored by iCELLigence™ real-time analysis (ACEA Biosciences) for five days.

For cell apoptosis assays, cells were seeded in 12-well plates at a concentration of 1 × 10^5^/well. Apoptosis was evaluated 72 h and 120 h post-transfection by detection of annexin V (APC) and propidium iodide (PI) by Cytoflex flow cytometer (Beckman Coulter Inc., Fullerton, California, USA) according to the manufacturer’s instructions.

### Cell cycle analysis

The cell cycle was analyzed as described previously^[29]^. Briefly, cells were cultured in the corresponding medium with reduced FBS (0.04%) at 2×10^5^ cells/6-well overnight. After transfection with either val-miR218 (20 nM) or NC miRNA (20 nM) and incubation for 48 h or 72 h, cells were harvested and washed with pre-cooled PBS and centrifuged for 10 min (4 °C, 400 g). Subsequently, cells were fixed by dropwise ice-cold 70% ethanol and continuous vortexing. The cells were washed and treated with RNase A, stained with PI, and evaluated on a Cytoflex flow cytometer (Beckman Coulter).

### RNA-Seq, pulldown-Seq, and bioinformatics

The val-miR218 mimics and NC miRNA were transfected to U2OS cells seeded in a 6-cm dish at a density of 1.2×10^6^ cells per dish. Total RNA was isolated using Trizol (Thermo Scientific) from two biological replicates 48 h after transfection.

Pulldown experiments with val-miR218 and NC miRNA were performed as described previously^[30]^. Biotinylated val-miR218 and biotinylated control miRNA were synthesized by Qiagen company (Hilden, Germany), annealed, and transfected into U2OS cells. The cells were collected 24 h post-transfection, washed with cold PBS, and incubated with lysis buffer (20 mM Tris with pH of 7, 25 mM EDTA, 100 mM KCl, 5 mM MgCl_2_, 0.3% NP-40, 50 U of RNase OUT and SUPERaseIn RNase Inhibitor [Thermo Scientific], and cOmplete protease inhibitor cocktail [Roche, Basel, Switzerland]) on ice for 20 min. After centrifugation at 10,000 g for 20 min, 50 μL cytoplasmic lysate was collected as an input sample and stored at -80 °C for further RNA isolation. The remaining cytoplasmic lysate was mixed with yeast tRNA and BSA (Thermo Scientific)-blocked streptavidin-coated magnetic beads (Dynabeads M-280, Thermo Scientific) and rotated at 4 °C for 4 h. The beads were washed five times with 1 mL lysis buffer, and bead-bound RNA was isolated using Trizol LS (Thermo Scientific).

Libraries were prepared using NEBNext Ultra RNA Library Prep Kit (New England Biolabs, Ipswich, Massachusetts, USA) and sequenced on the Illumina platform (Illumina, San Diego, CA, USA). Low-quality reads and reads with adapter contamination were discarded. The clean reads were aligned to the human genome by using HISAT2. The uniquely mapped reads were quantified using HTSeq. Differentially expressed genes were called using DESeq2, with the fold enrichment and p-value cutoffs at 1 and 0.05, respectively.

The transcripts found both in down-regulated genes after val-miR218 transfection and the genes enriched in biotinylated val-miR218 pulldown-seq were considered the most bona fide target candidates [Supplementary Table 1]. The algorithm TargetScan was also used to predict the targets [Supplementary Table 1].

### Target functional analysis

Ingenuity pathways analysis (IPA, Qiagen company, Hilden, Germany) was used to connect val-miR218 target candidates that were directly related to IPA database. The genes were subject to a core analysis with the default IPA settings to generate a list of IPA scores for the top associated network functions and significantly enriched molecular functions.

### RT-qPCR

Total RNA was isolated from transfected cells using Trizol (Thermo Scientific). According to the manufacturer's instructions, the first-strand cDNA synthesis was performed using a Maxima H minus First Strand cDNA Synthesis Kit (Thermo Scientific). Quantitative real-time PCR (qPCR) was conducted using the PowerUp SYBR green master mix (Thermo Scientific) and the PikoReal real-time PCR System (Thermo Scientific). The primer sequences and the real-time PCR reaction conditions used are listed in [Supplementary Table 2]. The melting curve was generated to test the specificity of PCR products and check for false-positive peaks. Transcript levels were normalized to the housekeeping gene GAPDH and analyzed by the relative quantification 2ˆ(-delta delta CT) method.

### Western blot

For protein expression, cells were incubated for 48 h after transfection with either val-miR218 (20 nM) or NC miRNA (20 nM). Afterward, cells were washed twice with PBS and lysed with RIPA buffer (Thermo Scientific) containing a cOmplete protease inhibitor cocktail. Protein concentration was determined using Bradford solution (Bio-Rad, Feldkirchen, Germany). Cell lysates (30 µg protein/lane) were separated on SDS-PAGE, transferred to nitrocellulose membranes (Bio-Rad), and blocked with 5 % non-fat milk in 50 mM Tris-buffered saline containing 0.05% Tween-20 (TBS-T) for 1 h at room temperature. Blots were incubated overnight at 4 °C in TBS-T containing 5 % BSA and primary antibody, washed thrice in TBS-T, and incubated 1 h with HRP-conjugated secondary antibodies (anti-rabbit and anti-mouse, Bio-Rad), then visualized by ECL (Thermo Scientific) on a Molecular Imager ChemiDoc (Bio-Rad). Primary antibodies that are directed against RPL12 (#A303-940A), RSP23 (#PA5-101372), RAB5C (#PA5-96646), BANF1 (#MA5-34813), WBP2 (#BS-11625R), NAE1 (#PA5-98379), Anillin (#A301-406A), RPIM2 (#A305-274A), PoIE3 (#A301-245A), GAPDH (#MA5-15738) were purchased from Thermo Scientific; anti-GADD45A (#9662), anti-p21^Waf1/Cip1^ (#2947) and anti-p27^KIP1^ (#3686) antibody antibodies were obtained from Cell Signaling Technology (Danvers, MN, USA); anti-CDC123 (#TA505693) antibody was purchased from Origene (Rockville, MD, USA), and anti-β-actin-peroxidase antibody was from (#A3854) Merck Millipore (Burlington, MA, USA).

### Dual-luciferase assay

The miRNA recognition elements (MREs) in the transcripts were predicted by RNAhybrid (https://bibiserv.cebitec.uni-bielefeld.de/rnahybrid/). Thirteen MREs were randomly selected [Supplementary Table 3], synthesized, and ligated into the 3′UTR region of the pmirGLO Dual-Luciferase Vector (Promega, Madison, WI, USA). The efficient insertion was confirmed by DNA sequencing (LGC Genomics GmbH, Berlin, Germany). For the dual-luciferase assays, 75 ng of constructed plasmid and 2.5 pmol of val-miR218 or negative control miRNA were transfected into cells in 48-well plates using Lipofectamine 3,000 (Thermo Scientific). The cells were harvested 24 h after transfection, and the activities of firefly and Renilla luciferases were assayed sequentially using a Dual-luciferase assay system (Promega).

### Mistletoe EV isolation and characterization

Mistletoe leaves growing on *Malus sylvestris* (L.) Mill were collected from Botanical Garden (Berlin, Germany) in February 2020. The leaves (5 g) were washed and grounded in 50 mL PBS buffer (pH 7.4), supplied with 1 mM DTT and complete protease inhibitor cocktail (Roche, Germany). The raw extract was centrifuged at 1,000 g for 10 min, 4,000 g for 20 min, and 15,000 g for 90 min to remove large fiber and cell debris. The supernatant was filtered through a 0.8 μm filter. The EVs were concentrated and purified from the filtrate using the OptiPrep cushion procedure and gradient density ultracentrifugation^[31]^. Briefly, the filtrate was added to the centrifuge tube with 2 mL of 60% iodixanol (Sigma-Aldrich, St. Louis, MO, USA) at the bottom to form a cushion layer. After spinning at 40,000 rpm (118,000 g) for 70 min using a Beckman Ti70 rotor, a volume of 3 mL from the bottom of the ultracentrifuge tubes was collected, resulting in a mixture of 40 % iodixanol. A discontinuous gradient was created by placing 3 mL of 5 % iodixanol solution at the bottom. Subsequently, underlay 3 mL of 10 % iodixanol, 3 mL of 20 % iodixanol, 3 mL of 40 % iodixanol solution containing mistletoe EVs, followed by 1 mL of 60 % iodixanol. After centrifuging the tubes in an SW41 Ti rotor at 36,000 rpm (164,000 g) for 18 h in Beckman Optima LE-80K ultracentrifuge (Beckman Coulter), gently collect 13 individual 1 mL fractions from the top of the tubes. The fractions 9-11 that were located within the density range of 1.13-1.20 g/mL were combined and washed by cushion ultracentrifugation in PBS and resuspended in PBS for further application.

Mistletoe EVs were placed on Formvar-Carbon coated grids (Electron Microscopy Sciences, Hatfield, PA, USA), stained with Uranyless (Electron Microscopy Sciences), and observed under scanning electron microscopy (Hi-tech, Tokyo, Japan) in TEM mode. TEM imaging was performed using 30 kV acceleration voltage. The size of EVs was measured by Nanoparticle Tracking Analysis NTA (Malvern Panalytical, Malvern, UK), and the protein concentration of EVs was determined by BCA assay (Thermo Scientific).

To investigate if mistletoe EVs could rescue the cytotoxicity of val-miR218, U2OS cells were seeded in 96-well plates at 1×104 cells per well and treated with mistletoe EVs (10 μg/mL) or negative control miRNA inhibitor (NC inhibitor, 50 nM) or val-miR218 inhibitor (50 nM) or the combinations, MTT assay was performed 72 h after the treatment. The inhibitors were miRCURY LNA miRNA inhibitors (Qiagen, Hilden, Germany).

### Confocal microscopy

The mistletoe EVs were stained with red fluorescent lipid dye PKH26 (Merck Millipore, Burlington, MA, USA), and the free dye was removed by ultracentrifugation (at 40,000 rpm for 70 min). Furthermore, FAM-labeled val-miR218 was transfected into mistletoe EVs using Exo-Fect siRNA/miRNA Transfection Kit (SBI System Biosciences, Palo Alto, CA, USA) according to the manufacturer’s instructions. After 24 h incubation with either stained EVs or the EVs transfected with FAM labeled val-miR218, U2OS cells were stained with Hoechst 33342 (Merck Millipore) and Alexa Fluor 594 -phalloidin (Abcam, Cambridge, United Kingdom) and imaged under a confocal microscope (SP8, Leica Microsystems, Wetzlar, Germany).

### Animal xenograft experiment

Eight-week-old female NOG mice (NOD.Cg-Prkdcscid Il2rgtm1Sug/JicTac; Taconic Biosciences GmbH, Germany) were housed in a pathogen-free facility in IVC (Tecniplast, Italy) cages and fed an autoclaved standard diet (Ssniff, Soest, Germany) with acidified drinking water ad libitum. SAOS-2 cells (1×10^6^) were subcutaneously injected in PBS:Matrigel (1:1) into the left flank of six mice per treatment or control group. Intra-tumoral injections with single-stranded val-miR218 (0.75 mg/kg) or vehicle were started with palpable tumors (day 15 post cell transplantation). Injections were applied at 2-3 different areas directly into the tumor (5 mm under the skin). Compound or vehicle was administered twice per week. Body weight was measured before each treatment, and mice were carefully monitored for health and symptoms of toxicity. Animals were sacrificed by cervical dislocation at the end of the experiment. Animal experiments were performed in accordance with the German Animal Welfare Act and all procedures were approved by local authorities (Landesamt für Gesundheit und Soziales, LAGeSo Berlin, Germany) under approval number A-0010/19 for preclinical tumor experiments.

### Statistics

All experiments were performed in three independent experiments, for which means ± standard errors are plotted in bar graphs. MiRNA treated groups were compared to NC miRNA treated groups using t-tests, while statistical comparisons among multiple groups were performed using one-way analysis of variance (ANOVA) followed by multiple post-hoc comparisons (*t-test*). All results with *P* ≤ 0.05 were considered significant.

## RESULTS

### Screening bioactive mistletoe miRNAs

**Figure 1:**
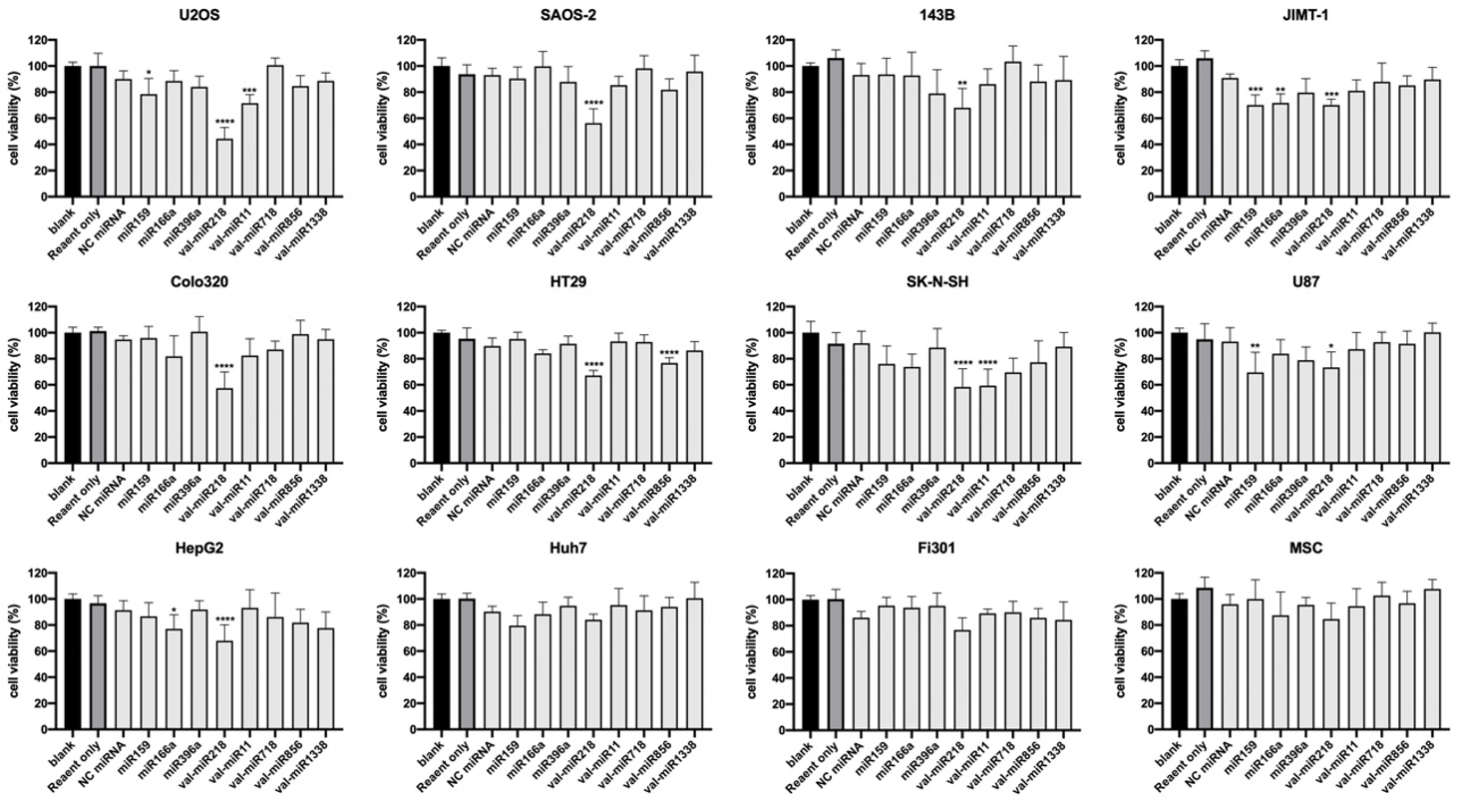
Bioactive mistletoe miRNAs screen for cytotoxic effects. Cells were seeded in 48-well plates at a density of 2 × 10^4^ cells per well and transfected with various miRNA mimics at a concentration of 20 nM. Cell viability was determined by MTT assay 72 h after transfection. *n* = 3. Compared with cells treated with NC miRNA, *P < 0.05; **P < 0.01; ***P < 0.001; **** *P* < 0.0001.

To analyze the bioactivity of mistletoe-specific miRNAs, three conserved miRNAs and five mistletoe-specific miRNAs with the most abundant expression were selected. These includes miR159, miR166a, miR396a, val-miR218, val-miR11, val-miR718, val-miR856, and val-miR1338. The miRNAs were transfected into the different tumor or non-tumor cells to check their cytotoxic effects. As shown in Figure 1, val-miR218 significantly reduced cell viability in most tested tumor cell lines, including osteosarcoma cell line U2OS, SAOS-2, 143B; breast carcinoma cell line JIMT-1; colon adenocarcinoma cell line Colo320R, HT-29; neuroblastoma cell line SK-N-SH; glioblastoma cell line U-87 and liver carcinoma cell line HepG2. Especially val-miR218 induced almost 50% viability in osteosarcoma cells U2OS and SAOS-2, respectively. However, there was no obvious toxicity of val-miR218 in human fibroblast cell line Fi301 and human mesenchymal stem cells (MSCs), indicating a great therapeutic potential of val-miR218.

In addition, some other miRNAs possessed cytotoxicity in specific cell lines too. Consistent with a previous report^[17]^, conserved miRNA miR159 inhibited the growth of JIMT-1, U2OS, and U-87 cells. Val-miR11 decreased cell viability in U2OS and SK-N-SH cells. But both miR159 and val-miR11 were less effective compared to val-miR218. Here in this study, we mainly focus on investigating the bioactive effects of val-miR218.

### Val-miR218 mimics strongly inhibited osteosarcoma cell growth and induced their apoptosis

**Figure 2:**
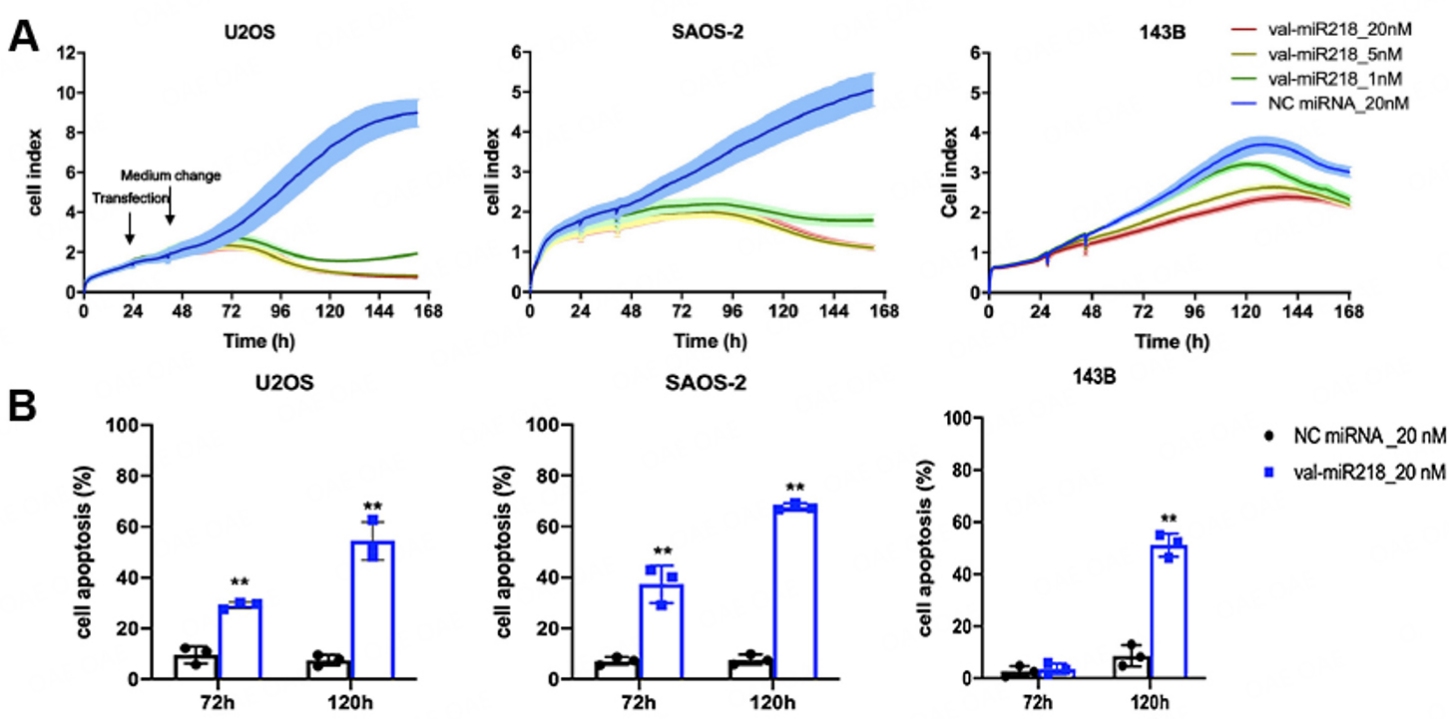
Val-miR218 inhibits cell proliferation and induces cell apoptosis in osteosarcoma cells in a time and concentration-dependent manner. (A) Osteosarcoma cells were seeded at a density of 2 × 10^4^ cells/ well in 8-well E-Plate (ACEA Biosciences, USA) and transfected with either val-miR218 or NC miRNA. The medium was changed 24 h after transfection. Cell proliferation was monitored by iCELLigence real-time analysis (ACEA Biosciences, USA) for a total of 7 days. (B) Cells were seeded at a density of 1×10^5^ cells/ well in 12-well plates and transfected with either val-miR218 or NC miRNA. Cell apoptosis was determined after depicted incubation periods by Annexin V/PI and FACS Flow cytometry. *n* = 3. Compared cells treated with NC miRNA, ** *P* < 0.01.

To evaluate the effect of val-miR218 on the proliferation of osteosarcoma cells U2OS, SAOS-2, and 143B, cells were transfected with val-miR218 and constantly monitored for 7 days. As shown in Figure 2A, the NC miRNA had no pronounced effect on cell proliferation, while val-miR218 mimics significantly inhibited proliferation in a time and concentration-dependent manner. For U2OS and SAOS-2, the cell index reduced up to 80% after val-miR218 transfection time-dependently [Figure 2A]. For 143B, a decrease in cell proliferation was observed, especially after 20 nM val-miR218 treatment compared to NC.

To determine the induction of apoptosis, val-miR218 treated cells were analyzed by annexin V/PI staining and FACS analysis after depicted time points [Supplementary Figure 1]. Apoptosis was enhanced with increased incubation time [Figure 2B]. SAOS-2 cells were more sensitive to val-miR218, which showed 10% more apoptotic cells in response to val-miR218, compared to U2OS cells.

For 143B, no prominent apoptosis was induced three days after transfection, but 50 % of cells were apoptotic 5 days after transfection.

### Identification of val-miR218 target transcripts

To obtain knowledge of the regulatory function of val-miR218 in osteosarcoma, U2OS cells were either transfected with biotinylated val-miR218 or biotinylated NC miRNA. The RNAs associated with biotinylated miRNAs were pulldown, sequenced, and analyzed. A total of 104 and 106 million uniquely mapped reads were obtained from two biological replicates from val-miR218 and control miRNA samples, respectively, of which about 94% were mapped to annotated transcripts. A total of 754 RNAs were found to be differently associated between biotinylated val-miR218 and biotinylated NC miRNA [cutoff conditions of Fold change > 1 and *P* value < 0.05, [Supplementary Table 1].

**Figure 3:**
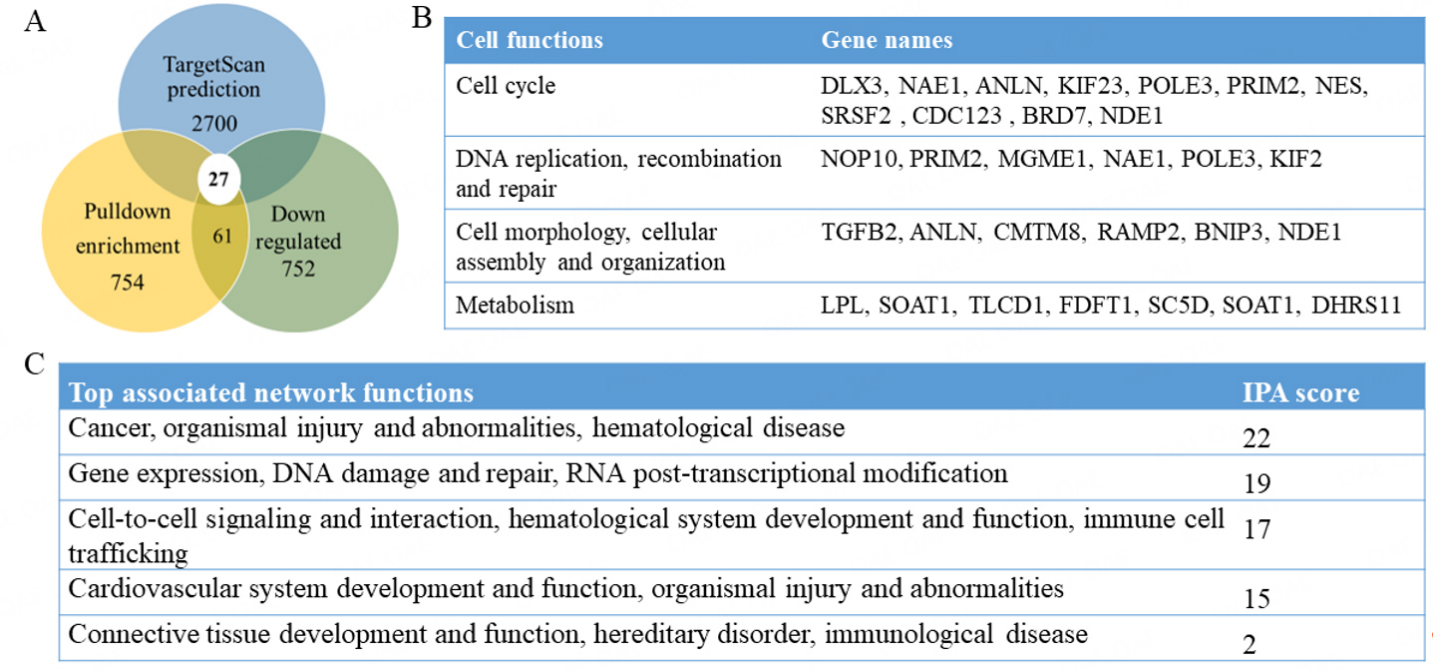
Target genes of val-miR218 and function analysis. (A) Venn diagram of val-miR218 target genes. (B) and (C) Molecular and cellular function, top associated network functions of “the most bona fide” val-miR218 targets by IPA (Qiagen).

The most bona fide RNA targets are diminished after overexpression of miRNA mimics^[30,32]^. To obtain these most bona fide mRNA targets, U2OS cells were transfected with val-miR218 or NC miRNA, and total RNA was isolated and sequenced on an Illumina platform. We obtained 49 million and 58 million uniquely aligned reads from two biological replicates, respectively. Compared to NC miRNA, val-miR218 mimics downregulated 752 genes [Fold change < 1 and *P* value < 0.05; Figure 3A; Supplementary Table 1]. To clarify how many down-regulated target genes are directly regulated by val-miR218 or indirectly regulated, the down-regulated genes were compared to biotinylated val-miR218 associated genes. As a result, an overlap of 61 target genes was identified. They were considered the most bona fide target candidates directly regulated by val-miR218 [Figure 3A; Supplementary Table 1].

We also used TargetScan as a bioinformatics algorithm to predict the val-miR218 targets. 27 of the 61 bona fide target genes were predicted by TargetScan, too [Supplementary Table 1].

**Figure 4:**
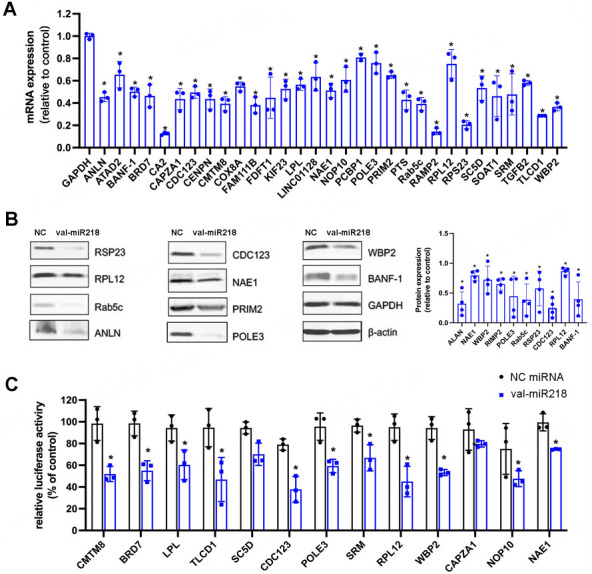
Most val-miR218 targets have reduced mRNA and protein expression after val-miR218 overexpression. (A) Val-miR218 target gene expression after val-miR218 overexpression in U2OS cells. The relative mRNA expression of val-miR218 treated cells was compared to those treated with NC miRNA. The relative mRNA expression of NC miRNA treated group was set as 1. *n* = 3. (B) Representative immunoblots of U2OS cells transfected with val-miR218 or NC miRNA. GAPDH and β-actin are housekeeping genes. The protein of interest was first adjusted to housekeeping proteins. Then, the relative protein expression of val-miR218 treated cells was compared to those treated with NC miRNA. The relative protein expression of NC miRNA treated group was set as 1. *n* = 4. (C) Dual-luciferase assay of 13 randomly selected miRNA recognition elements (MRE). Normalized to the luciferase activity of cells transfected with the corresponding vector only. *n* = 3. Compared cells treated with NC miRNA, * *P* < 0.05.

To evaluate the authenticity of the bona fide target candidates, 31 genes were randomly selected from the 61 target candidates and determined by quantitative RT-PCR, and 10 target protein expressions were examined by Western blot [Figure 4, Supplementary Figure 2]. As shown in Figure 4A, the RNA expression of these 31 targets was downregulated 48 h after val-miR218 transfection, which confirmed the RNA-seq data. The protein expression of the 10 selected transcripts was decreased in U2OS cells after val-miR218 overexpression [Figure 4]. These results indicated that val-miR218 is connected to these target genes and diminished their expression.

To confirm the direct interactions between val-miR218 and these target genes, we cloned the MREs of 13 target genes into dual-luciferase vectors. U2OS cells were co-transfected with constructed dual-luciferase vector together with val-miR218 or NC miRNA and checked for luciferase activity. As shown in Figure 4C, 11 of 13 MREs were significantly blocked by val-miR218, indicating val-miR218 recognized these transcripts and inhibited their expression via binding to their MREs.

### Val-miR218 target functional analysis

To reveal the function of val-miR218, we used IPA to predict the biological interactions and functions of genes. The interactome of 61 targets was analyzed to identify their molecular and cellular function, and top associated network functions. Among these 61 genes, 11 genes are related to cell cycle, 6 genes are related to DNA replication, recombination and repair, 6 genes are related to cell morphology, cellular assembly and organization, and 7 genes are related to lipid metabolism [Figure 3B]. The top associated network functions are listed in Figure 3C. Interestingly, the network function with the highest IPA score is associated with cancer, organismal injury and abnormalities, which fits our current study.

**Figure 5:**
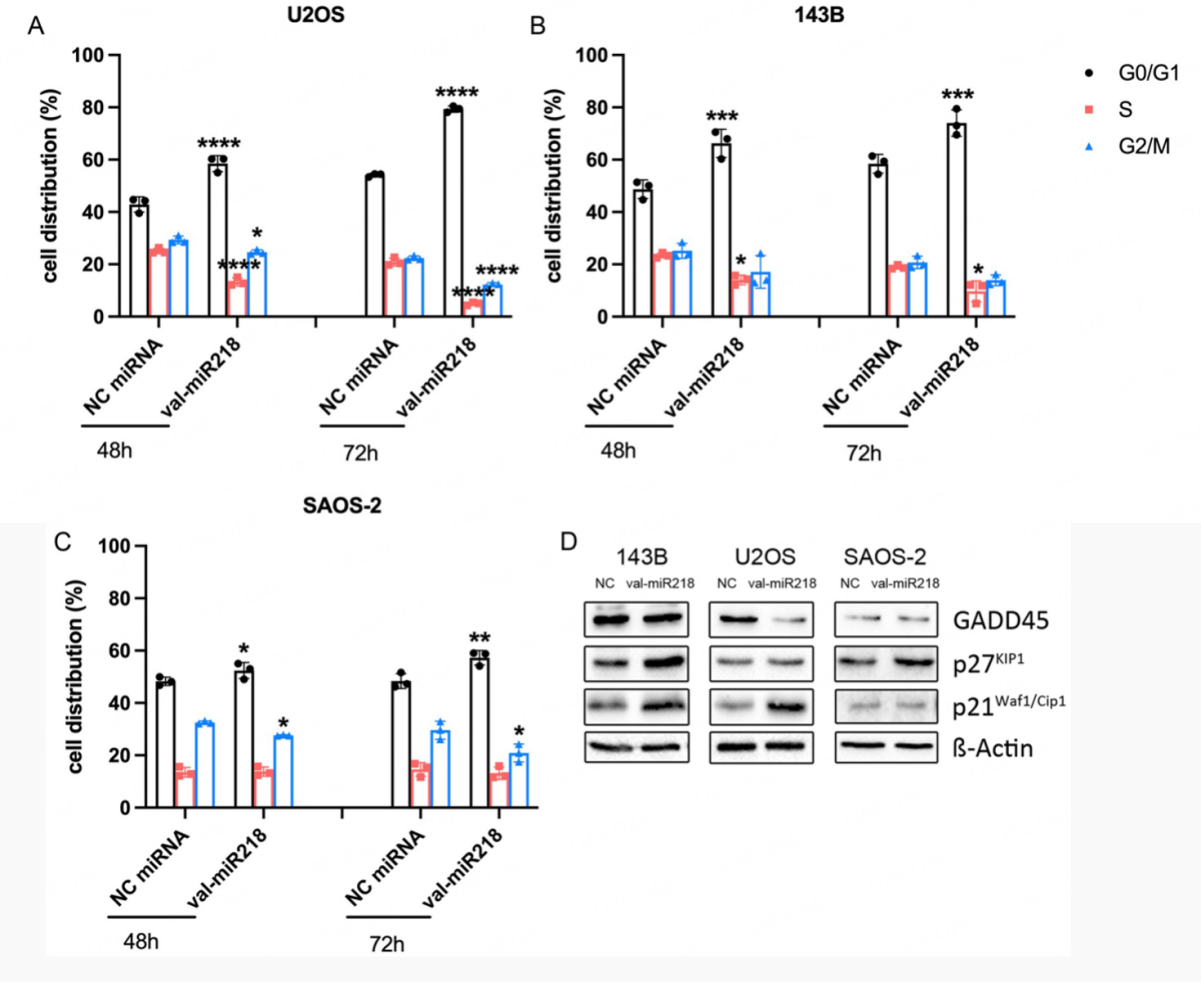
Effect of val-miR218 on cell cycle and related protein expression. (A) U2OS, (B) 143B and (C) SAOS-2 cells were seeded into 6-well plates at 2 × 10^5^ cells per well and serum-starved for 24 h. Cells were transfected with NC miRNA or val-miR218 and re-feeded with FBS. The cell cycle was analyzed either 48 h or 72 h after transfection using PI and FACS Flow cytometry analysis. *n* = 3. (D) Cells were seeded into 6 cm dishes and transfected with NC miRNA or val-miR218; protein was extracted 48 h after transfection and analysis for desired protein expression by western blot. *n* = 3. Compared cells treated with NC miRNA, *P < 0.05, **P < 0.01, ***P < 0.001, ****P < 0.0001.

The most significantly enriched function is the cell cycle, for which 11 target genes were annotated. Therefore, we investigated the effect of val-miR218 on the cell cycle [Supplementary Figure 3]. Val-miR218 overexpression significantly increased the number of cells in G0/G1 phase, especially in U2OS, 143B and SAOS-2 cells, time-dependently [Figure 5]. Moreover, val-miR218 overexpression led to an upregulation of p21^WAF1/CIP1^ in 143B and U2OS cells and p27 in all three cell lines after 48 h, which confirms the G1 phase arrest [Figure 5D, Supplementary Figure 4].

### Mistletoe EVs carry and transport val-miR218 to recipient cells

**Figure 6:**
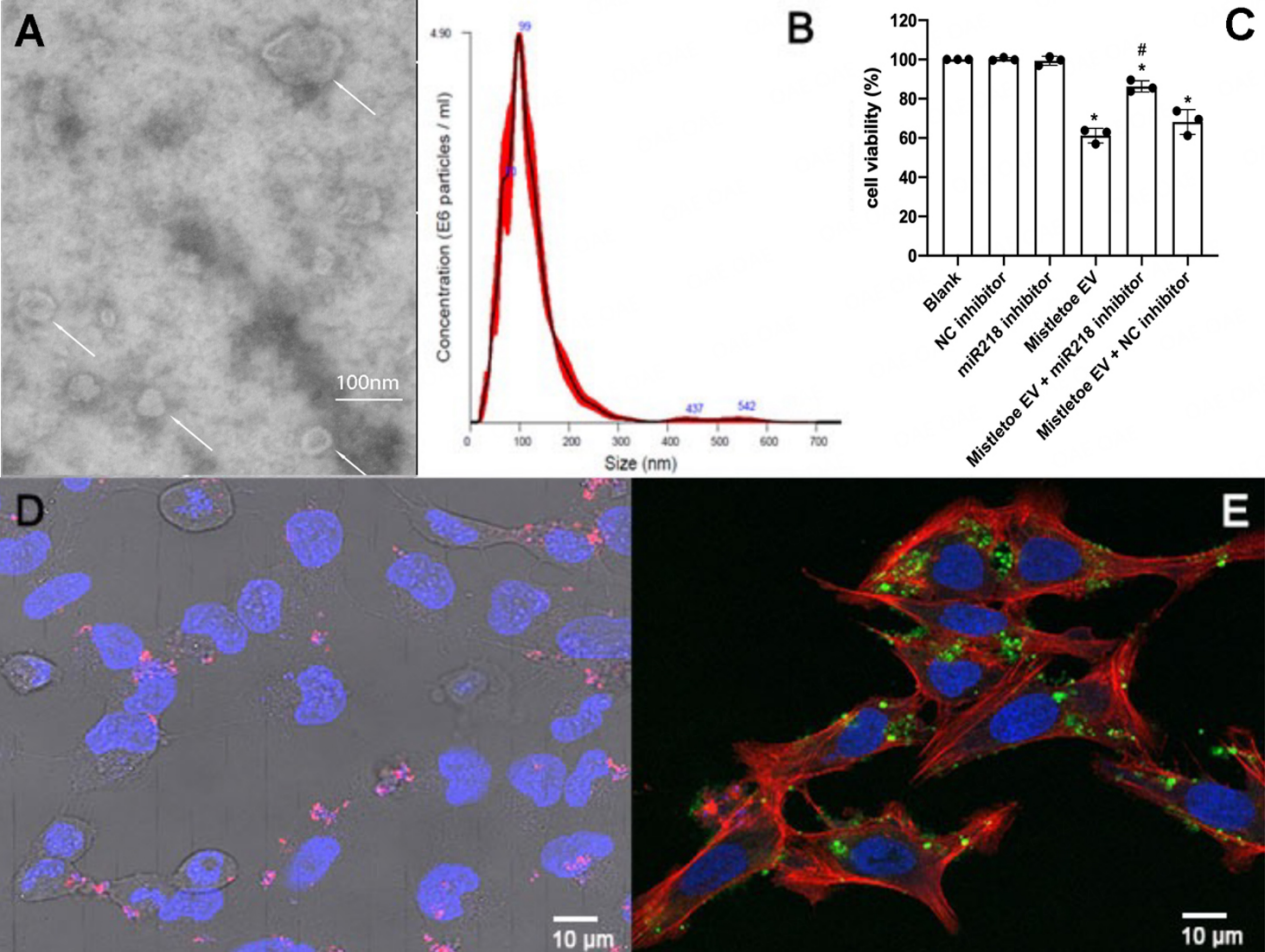
Mistletoe EVs might mediate cell uptake of val-miR218. (A) Visualization of mistletoe EVs under TEM. (B) Size distribution of mistletoe EVs. (C) U2OS cells were seeded in 96-well plates at 1 × 10^4^ cells per well and treated with mistletoe EVs (10 μg/mL) or negative control miRNA inhibitor (NC inhibitor, 50 nM) or val-miR218 inhibitor (50 nM) or the combinations, MTT assay was performed 72 h after the treatment. Compared with blank cells, *P < 0.05; compared with cells treated with EVs, #*P* < 0.05. (D) U2OS cells were incubated with PKH26 stained mistletoe EVs for 24 h, and cell uptake of EVs was checked under a confocal microscope. (E) FAM-labeled val-miR218 was transfected into EVs and then incubated with U2OS cells for 24 h. The interaction of EVs and cells was checked under a confocal microscope. The cell nucleus was stained by Hoechst 33342, and the cell skeleton was stained by phalloidin. *n* = 3.

We next wanted to evaluate whether mistletoe EVs mediate the function of val-miR218. Therefore, mistletoe EVs were isolated from mistletoe extracellular fluid using gradient density ultracentrifugation. They showed typical round and cup-shaped structures under SEM, with an average diameter of 119 nm [Figure 6A and B]. To evaluate if mistletoe EVs mediate the function of val-miR218, U2OS cells were either incubated with mistletoe EVs alone or in combination with val-miR218 inhibitor, negative control miRNA inhibitor, respectively. The incubation with mistletoe EVs caused a 40% reduction in cell viability. As shown in Figure 6C, the NC inhibitor did not influence the effect of mistletoe EVs, whereas the val-218 inhibitor significantly rescued the EV-induced cytotoxicity. However, val-miR218 inhibitors did not completely inhibit the impact of EVs; around 15% of cells were still found dead in the cells co-treated with EVs and val-miR218.

To check if mistletoe EVs transport val-miR218 into mammalian cells, we first labeled EVs with red fluorescent lipid dye PKH26 and incubated them with U2OS cells. As shown in Figure 5D, EVs were successfully taken by U2OS cells after 24 h incubation. Furthermore, FAM-labeled val-miR218 was transfected into mistletoe EVs. After 24 h incubation with EVs, the uptake of val-miR218 by U2OS was directly observed under confocal microscope (SP8, Leica Microsystems, Germany). However, without the assistant EVs, FAM labeled val-miR218 could not be taken up by U2OS cells [Supplementary Figure 5]. The data suggest that mistletoe EVs facilitate the uptake of val-miR218 by U2OS cells. However, additional research is required to evaluate whether mistletoe EVs can also mediate the uptake of val-miR218 in other cell lines.

### Val-miR218 reduces tumor growth *in vivo*.

**Figure 7:**
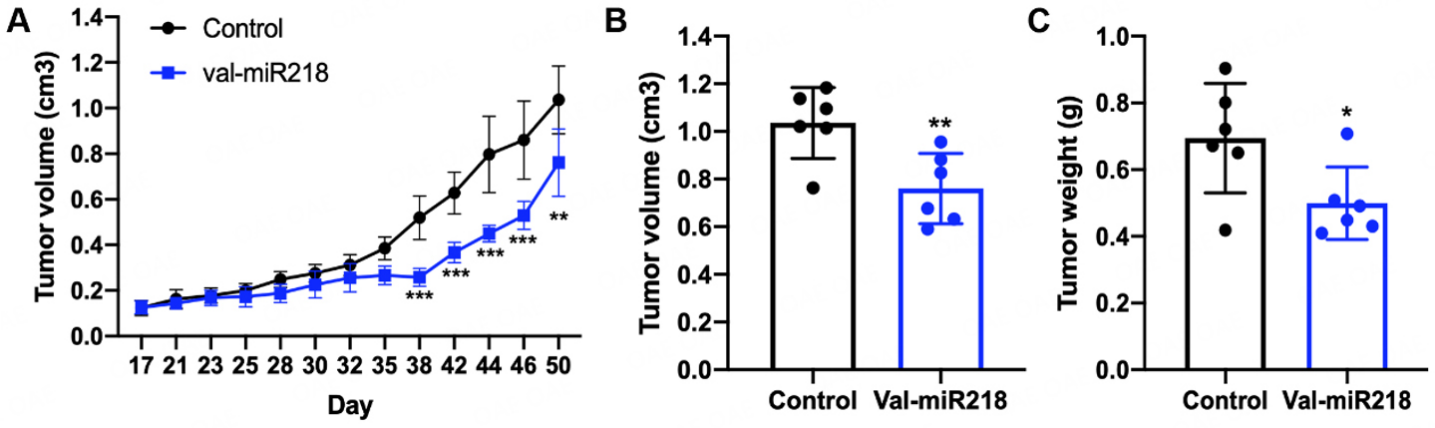
Val-miR218 reduces tumor weight and tumor volume *in vivo.* Xenografts were treated intratumorally with 0.75 mg/kg of val-miR218 twice weekly. The application was given in a total of 9 times. (A) Tumor volume was measured every 3 days. (B) and (C) Tumor volume and tumor weight were determined one day after the last injection. Compared to control group, *P<0.05, **P<0.01, ***P<0.001.

To investigate the therapeutic potential of val-miR218, the efficacy was tested *in vivo*. For this purpose, Saos-2 xenografts were intratumorally treated nine times for approximately four weeks with single-stranded val-miR218. Val-miR218 treatment led to a significant reduction in tumor weight and tumor volume in comparison to the control group [Figure 7]. Mice did not show significant changes in body weight or signs of toxicity during the study, which means the val-miR218 treatment was well tolerated. These data indicated val-miR218 as a promising miRNA for tumor treatment *in vivo.* The underlying mechanism will be further investigated in our future studies.

## DISCUSSION

Recent research has revealed the presence of intact and functional plant-derived miRNAs in human fluids and tissues, suggesting possible cross-kingdom regulatory effects^[15-21]^. The idea of cross-kingdom and gene regulation in humans by plant miRNAs serves as a promising treatment strategy for treating malignant tumors.

Osteosarcoma is a rare but highly prevalent form of primary bone cancer. In the USA, approximately 1,000 new cases are reported annually, with half of them affecting children and adolescents^[33]^. Current treatment for osteosarcomas involves surgery combined with chemotherapy. However, high-grade osteosarcoma patients may develop pulmonary metastasis, which significantly reduces their chances of recovery. Moreover, conventional therapies can result in chemotherapy resistance, making novel adjuvant therapy essential^[33]^.

As the European mistletoe has been widely used for the adjuvant treatment of cancer, we analyzed the occurrence of mistletoe-specific miRNAs in one of our previous studies^[25]^. Interestingly, these mistletoe-specific miRNAs are predicted to be associated with human diseases, including anti-cancer effects and prevention of cardiovascular and neurological disorders^[25]^. The stability of some mistletoe miRNAs in various mistletoe preparations has been proven in our subsequent study^[26]^. Here, we identified bioactive miRNAs from mistletoe and confirmed the anti-cancer effects of val-miR218 both *in vitro* and *in vivo* for the first time.

Three conserved miRNAs and five mistletoe-specific miRNAs with the most abundant expression were tested for their anti-tumor effect *in vitro*. As the most abundant mistletoe-specific miRNAs, val-miR218 showed significant cytotoxicity in most tested tumor cells, especially osteosarcoma U2OS, SAOS-2, and 143B cells. In addition, val-miR218 effectively inhibited osteosarcoma cell proliferation and induced cell apoptosis time-dependently, indicating its promising potential for cancer treatment therapy. Notably, val-miR218 exhibited a more substantial anti-tumor effect than miR159, a vegetable-derived miRNA that is “well-known” for its anti-breast cancer effect^[17]^.

RNA-seq was performed to elucidate the targets of val-miR218. As a result, 61 transcripts were considered as direct targets of val-miR218 since they were found both in the list of biotin-labeled val-miR218 associated RNAs and the list of val-miR218 down-regulated genes. Of the 61 candidate targets, 27 were also predicted by TargetScan. These targets have been confirmed by quantitative RT-PCR and western blot, and the MREs have been validated by dual-luciferase assay [Figure 3]. Remarkably, these direct targets are closely related to essential cell functions, including cell cycle, DNA replication, recombination and repair, cell morphology, cellular assembly and organization, and lipid metabolism. Specifically, 11 of these 61 targets are involved in the cell cycle; for instance, PRIM2 and POLE3 are necessary for DNA replication initiation during the S phase of the cell cycle^[34-36]^. SRSF2 and CDC123 are required for S phase entry of the cell cycle; inhibition of SRSF2 or CDC123 causes G1 cell cycle arrest and G2 delay^[37,38]^. NAE1 is necessary for cell cycle progression through the S-M checkpoint^[39,40]^. ANLN and KIF23 are involved in cell division in the M phase^[41,42]^. NDE1 and NES are required in the G2/M transition of the mitotic cell cycle^[43,44]^. The protein expression of ANLN, CDC123, NAE1, PRIM2, and POLE3 has been experimentally confirmed [Figure 3B]. By down-regulating of these proteins, val-miR218 strongly affected cell cycle progress and inhibited cell proliferation. Additionally, WBP2 (WW domain-binding protein 2) is an emerging oncogene involved in Wnt, EGFR, Hippo, and PI3K /Akt pathways^[45]^.It was reported that inhibition of WBP2 expression by miR-613 repressed breast cancer growth^[46]^. Even the role of WBP2 in osteosarcoma remains clarified; the downregulation of WBP2 by val-miR218 might contribute to its inhibitory effect in osteosarcoma.

The overexpression of val-miR218 increased the p21WAF1/CIP1 protein level in 143B cells, especially in the p53 wild-type cell line U2OS. This leads to the assumption that the transcription factor p53 enhances p21WAF1/CIP1 transcription at the G1 phase, inhibiting cyclin-dependent kinase activity and leading to G1 arrest^[47-50]^. P27, another key regulator of cell proliferation, shows an upregulation too. It can bind and inhibit the cyclin E/Cdk2 and cyclin D/CDK4 complexes at the G1 phase and halts cell cycle progression. The mode of action of val-miR218 needs to be further evaluated.

It is well known that naked miRNAs are extremely sensitive in herb preparations and the mammalian circulation system. Our previous study detected val-miR218 in various mistletoe extracts and indicated the relative stability of val-miR218^[26]^. Mistletoe preparations are usually applied via subcutaneous injection^[27,28]^. Being packed into EVs, mistletoe miRNAs could be transferred to the circulating system or directly reach receipt cells via EVs. In the present study, val-miR218 inhibitors could significantly reverse mistletoe EV-induced cytotoxicity, indicating val-miR218 is associated with mistletoe EVs. Being loaded inside EVs, val-miR218 could be efficiently delivered into human cells. It is also noteworthy that val-miR218 inhibitors could not wholly block the cytotoxic effect of mistletoe EVs, indicating there might be other active miRNAs (e.g., miR159 and val-miR11 that also showed cytotoxicity in U2OS cells), or mistletoe lectins or viscotoxins might be packaged into the EVs. Further studies are needed to identify the miRNA and protein profile in mistletoe EVs. The mistletoe EVs offer not only an efficient vehicle but also a naturally derived combination of various bioactive ingredients.

In conclusion, we have demonstrated the high potential of val-miR218 in inhibiting proliferation in various tumor cell lines *in vitro*, as well as for osteosarcoma *in vivo*, for the first time. We identified 61 direct targets of val-miR218, which are related to essential cellular functions such as cell cycle, DNA replication, and cell morphology. Furthermore, we confirmed the downregulation of selected targets on both mRNA and protein levels. Given the increasing problems during chemotherapy, such as resistance or negative side effects, new therapeutic approaches are becoming more critical. The widespread triggering of proliferation inhibition targets indicates a promising therapeutic strategy for treating cancer.

The study of cross-kingdom regulation of exogenous plant miRNAs is currently at an early stage. The basis of the clinical application of exogenous miRNAs lies in their exceptional stability in harsh gastrointestinal or blood environments. However, the development of chemical modifications (e.g., the addition of a 2’-O-methyl group or locked nucleic acids) and delivery systems (e.g., neutral lipid emulsions, plant or human cell-derived EVs) for miRNA mimics and antimiRs remains a crucial challenge for their *in vivo* applications^[10]^.

## LIMITATIONS OF THIS STUDY

It is essential to acknowledge the potential implications of the limitations. In our study, we have successfully isolated intact EVs from mistletoe and demonstrated their ability to transport val-miR218 to U2OS cells. Specifically, we isolated the EVs from winter-grown mistletoe of *Malus sylvestris* (L.) Mill, and it is known that the proportion of pharmacologically relevant substances in *V. album* varies depending on the host tree and collection season^[51]^. However, it remains unclear whether seasonal differences influence the composition of mistletoe-derived EVs and how this might affect their ability to transport cargo. Due to our low yield of EVs from the limited harvested material, we selected p53 wild-type U2OS cells for our initial experiment since they are highly sensitive to val-miR218. Unfortunately, we could not perform additional experiments with SAOS-2 and 143B cell lines due to the limited number of EVs isolated from winter-grown mistletoe. Another limitation of this study is the lack of known surface markers for mistletoe-derived EVs. Although a few plant EV markers have been discovered in selected plants, such as PEN1, PEN3, and TET8 in *A. thaliana*^[52]^, and HSP70, GAPDH, and S-adenosyl-homocysteine in *A. thaliana*, *N. benthamiana*, and sunflower^[53]^, respectively, ideal “universal” plant EV markers still need to be identified. Therefore, we used traditional methods, including nanoparticle tracking and TEM, to determine the size and morphology of mistletoe EVs, as previously described^[54]^. Future research is required to develop such universal plant EV markers.

Another aim of this study was to investigate the regulatory role of val-miR218 in osteosarcoma and to identify target genes. We successfully identified and validated target transcripts of val-miR218 in the U2OS cell line. However, to further strengthen our findings, validation of these targets in additional cell lines such as SAOS-2 and 143B would be beneficial. This would provide a more comprehensive understanding of how val-miR218 affects gene translation in various cell lines with different (p53) mutation levels. Therefore, future studies will focus on this aspect.

## DECLARATIONS

### Availability of data and materials

The data that support the findings of this study are available from the corresponding author upon reasonable request.

### Copyright

© The Author(s) 2023.

## References

[1] Zhu Y, Zhu L, Wang X, Jin H (2022). RNA-based therapeutics: an overview and prospectus. Cell Death Dis.

[2] Nielsen AF, Bindereif A, Bozzoni I (2022). Best practice standards for circular RNA research. Nat Methods.

[3] Voinnet O (2009). Origin, biogenesis, and activity of plant microRNAs. Cell.

[4] Friedman RC, Farh KK, Burge CB, Bartel DP (2009). Most mammalian mRNAs are conserved targets of microRNAs. Genome Res.

[5] Chen X, Rechavi O (2022). Plant and animal small RNA communications between cells and organisms. Nat Rev Mol Cell Biol.

[6] Li C, Ni YQ, Xu H (2021). Roles and mechanisms of exosomal non-coding RNAs in human health and diseases. Signal Transduct Target Ther.

[7] Sohel MH (2016). Extracellular/circulating micrornas: release mechanisms, functions and challenges. Achievements in the Life Sciences.

[8] Mori MA, Ludwig RG, Garcia-Martin R, Brandão BB, Kahn CR (2019). Extracellular miRNAs: from biomarkers to mediators of physiology and disease. Cell Metab.

[9] Makarova J, Turchinovich A, Shkurnikov M, Tonevitsky A (2021). Extracellular miRNAs and cell-cell communication: problems and prospects. Trends Biochem Sci.

[10] Rupaimoole R, Slack FJ (2017). MicroRNA therapeutics: towards a new era for the management of cancer and other diseases. Nat Rev Drug Discov.

[11] Diener C, Keller A, Meese E (2022). Emerging concepts of miRNA therapeutics: from cells to clinic. Trends Genet.

[12] Moran Y, Agron M, Praher D, Technau U (2017). The evolutionary origin of plant and animal microRNAs. Nat Ecol Evol.

[13] Wynant N, Santos D, Vanden Broeck J (2017). The evolution of animal Argonautes: evidence for the absence of antiviral AGO Argonautes in vertebrates. Sci Rep.

[14] Singh RK, Gase K, Baldwin IT, Pandey SP (2015). Molecular evolution and diversification of the Argonaute family of proteins in plants. BMC Plant Biol.

[15] Zhang L, Hou D, Chen X (2012). Erratum: exogenous plant MIR168a specifically targets mammalian LDLRAP1: evidence of cross-kingdom regulation by microRNA. Cell Res.

[16] Zhou Z, Li X, Liu J (2015). Honeysuckle-encoded atypical microRNA2911 directly targets influenza A viruses. Cell Res.

[17] Chin AR, Fong MY, Somlo G (2016). Cross-kingdom inhibition of breast cancer growth by plant miR159. Cell Res.

[18] Chen X, Liu L, Chu Q (2021). Large-scale identification of extracellular plant miRNAs in mammals implicates their dietary intake. PLoS One.

[19] Zhou LK, Zhou Z, Jiang XM (2020). Absorbed plant MIR2911 in honeysuckle decoction inhibits SARS-CoV-2 replication and accelerates the negative conversion of infected patients. Cell Discov.

[20] Gismondi A, Nanni V, Monteleone V, Colao C, Di Marco G, Canini A (2021). Plant miR171 modulates mTOR pathway in HEK293 cells by targeting GNA12. Mol Biol Rep.

[21] Zhang S, Sang X, Hou D (2019). Plant-derived RNAi therapeutics: a strategic inhibitor of HBsAg. Biomaterials.

[22] Li M, Chen T, Wang R

[23] Li M, Chen T, He JJ (2019). Plant MIR167e-5p inhibits enterocyte proliferation by targeting β-catenin. Cells.

[24] Horneber MA, Bueschel G, Huber R, Linde K, Rostock M (2008). Mistletoe therapy in oncology. Cochrane Database Syst Rev.

[25] Xie W, Adolf J, Melzig MF (2017). Identification of viscum album L. miRNAs and prediction of their medicinal values. PLoS One.

[26] Xie W, Melzig MF (2018). The Stability of medicinal plant microRNAs in the herb preparation process. Molecules.

[27] Steele ML, Axtner J, Happe A, Kröz M, Matthes H, Schad F (2015). Use and safety of intratumoral application of European mistletoe (Viscum album L) preparations in Oncology. Integr Cancer Ther.

[28] Marvibaigi M, Supriyanto E, Amini N, Abdul Majid FA, Jaganathan SK (2014). Preclinical and clinical effects of mistletoe against breast cancer. Biomed Res Int.

[29] Kleinsimon S, Longmuss E, Rolff J (2018). GADD45A and CDKN1A are involved in apoptosis and cell cycle modulatory effects of viscumTT with further inactivation of the STAT3 pathway. Sci Rep.

[30] Tan SM, Kirchner R, Jin J (2014). Sequencing of captive target transcripts identifies the network of regulated genes and functions of primate-specific miR-522. Cell Rep.

[31] Zhuang X, Deng Z-B, Mu J (2015). Ginger-derived nanoparticles protect against alcohol-induced liver damage. J Extracell Vesicles.

[32] Lal A, Thomas MP, Altschuler G (2011). Capture of microRNA-bound mRNAs identifies the tumor suppressor miR-34a as a regulator of growth factor signaling. PLoS Genet.

[33] Mirabello L, Troisi RJ, Savage SA (2009). International osteosarcoma incidence patterns in children and adolescents, middle ages and elderly persons. Int J Cancer.

[34] Weiner BE, Huang H, Dattilo BM, Nilges MJ, Fanning E, Chazin WJ (2007). An iron-sulfur cluster in the C-terminal domain of the p58 subunit of human DNA primase. J Biol Chem.

[35] Wang T, Tang T, Jiang Y (2022). PRIM2 promotes cell cycle and tumor progression in p53-mutant lung cancer. Cancers.

[36] Xu X, Duan S, Hua X, Li Z, He R, Zhang Z (2022). Stable inheritance of H3.3-containing nucleosomes during mitotic cell divisions. Nat Commun.

[37] Edmond V, Merdzhanova G, Gout S, Brambilla E, Gazzeri S, Eymin B (2013). A new function of the splicing factor SRSF2 in the control of E2F1-mediated cell cycle progression in neuroendocrine lung tumors. Cell Cycle.

[38] Panvert M, Dubiez E, Arnold L (2015). Cdc123, a cell cycle regulator needed for eIF2 assembly, is an ATP-grasp protein with unique features. Structure.

[39] Chen Y, McPhie DL, Hirschberg J, Neve RL (2000). The amyloid precursor protein-binding protein APP-BP1 drives the cell cycle through the S-M checkpoint and causes apoptosis in neurons. J Biol Chem.

[40] Olaizola P, Lee-Law PY, Fernandez-Barrena MG (2022). Targeting NAE1-mediated protein hyper-NEDDylation halts cholangiocarcinogenesis and impacts on tumor-stroma crosstalk in experimental models. J Hepatol.

[41] Magnusson K, Gremel G, Rydén L (2016). ANLN is a prognostic biomarker independent of Ki-67 and essential for cell cycle progression in primary breast cancer. BMC Cancer.

[42] Fischer M, Grundke I, Sohr S (2013). p53 and cell cycle dependent transcription of kinesin family member 23 (KIF23) is controlled via a CHR promoter element bound by DREAM and MMB complexes. PLoS One.

[43] Wang Q, Wu H, Hu J (2021). Nestin Is required for spindle assembly and cell-cycle progression in glioblastoma cells. Mol Cancer Res.

[44] Kim S, Zaghloul NA, Bubenshchikova E (2011). Nde1-mediated inhibition of ciliogenesis affects cell cycle re-entry. Nat Cell Biol.

[45] Tabatabaeian H, Rao A, Ramos A, Chu T, Sudol M, Lim YP (2020). The emerging roles of WBP2 oncogene in human cancers. Oncogene.

[46] Song H, Wu T, Xie D (2018). WBP2 downregulation inhibits proliferation by blocking YAP Transcription and the EGFR/PI3K/Akt signaling pathway in triple negative breast cancer. Cell Physiol Biochem.

[47] Barr AR, Cooper S, Heldt FS (2017). DNA damage during S-phase mediates the proliferation-quiescence decision in the subsequent G1 via p21 expression. Nat Commun.

[48] Deng C, Zhang P, Harper JW, Elledge SJ, Leder P (1995). Mice lacking p21CIP1/WAF1 undergo normal development, but are defective in G1 checkpoint control. Cell.

[49] Shen G, Xu C, Chen C, Hebbar V, Kong AN (2006). p53-independent G1 cell cycle arrest of human colon carcinoma cells HT-29 by sulforaphane is associated with induction of p21CIP1 and inhibition of expression of cyclin D1. Cancer Chemother Pharmacol.

[50] Jeong JH, Kang SS, Park KK, Chang HW, Magae J, Chang YC (2010). p53-independent induction of G1 arrest and p21WAF1/CIP1 expression by ascofuranone, an isoprenoid antibiotic, through downregulation of c-Myc. Mol Cancer Ther.

[51] Nazaruk J, Orlikowski P (2016). Phytochemical profile and therapeutic potential of Viscum album L. Nat Prod Res.

[52] Rutter BD, Innes RW (2020). Growing pains: addressing the pitfalls of plant extracellular vesicle research. New Phytol.

[53] Pinedo M, de la Canal L, de Marcos Lousa C (2021). A call for Rigor and standardization in plant extracellular vesicle research. J Extracell Vesicles.

[54] Woith E, Melzig MF (2019). Extracellular vesicles from fresh and dried plants-simultaneous purification and visualization using gel electrophoresis. Int J Mol Sci.

