# Supplementary material for: Inhibition of osteosarcoma by European Mistletoe derived val-miR218
Source: Extracell Vesicles Circ Nucl Acids. 2023 Jul 3;4(3):306–22. doi: 10.20517/evcna.2023.15 (PMC11651123; doi:10.20517/evcna.2023.15)
Supplement: Supplementary file 2 [file evcna-4-3-306-SupplementaryMaterials.pdf]

Supplementary Materials

Inhibition of osteosarcoma by European Mistletoe derived val-miR218

Wenyan Xie[1,*], Catharina Delebinski[2], Dennis Gürgen[3], Maik Schröder[2], Georg Seifert[2,#], Matthias F. Melzig[1,#]

[1]Institute of Pharmacy, Freie Universität Berlin, Berlin 14195, Germany.
[2]Department of Pediatric Oncology/Hematology, Otto-Heubner-Centre for Pediatric and Adolescent Medicine (OHC), Charité – Universitätsmedizin Berlin, corporate member of Freie Universität Berlin and Humboldt Universität zu Berlin, Berlin 13353, Germany.
[3]EPO GmbH Berlin, Berlin 13353, Germany.

[#]Authors contributed equally.

***Correspondence to:** Dr. Wenyan Xie, Institute of Pharmacy, Freie Universität Berlin, Königin-Luise-Str. 2+4, 14195 Berlin, Germany. E-mail: xiewenyanyan@yahoo.com

ORCID: Wenyan Xie (0000-0001-6898-3216)

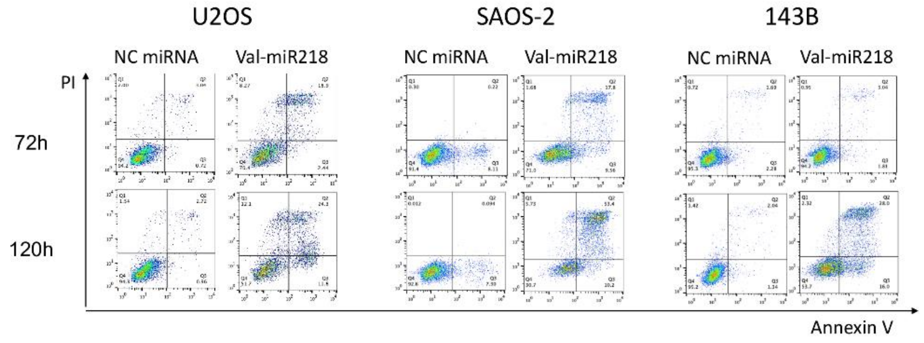

Supplementary Figure 1. FACS analysis of cell apoptosis.

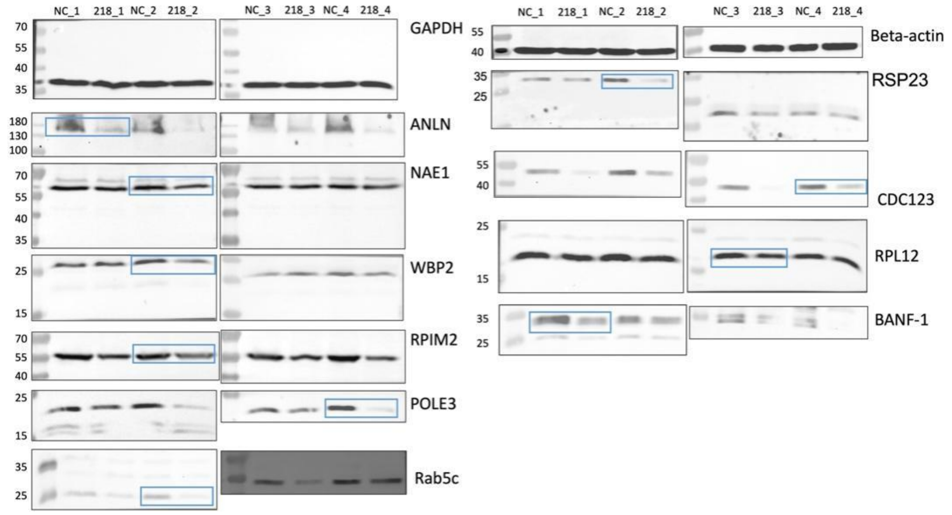

Supplementary Figure 2. Western blot images of U2OS cells transfected with val-miR218 or NC miRNA. The blots in blue frame are also present in Figure 4.

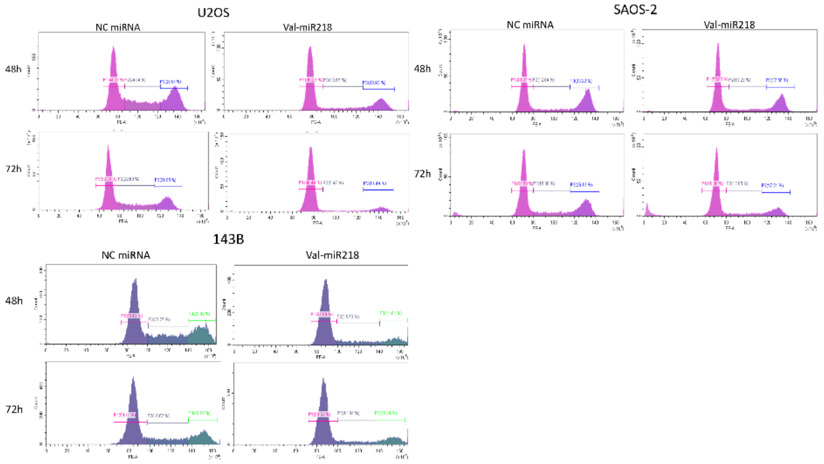

Supplementary Figure 3. FACS analysis of cell cycle.

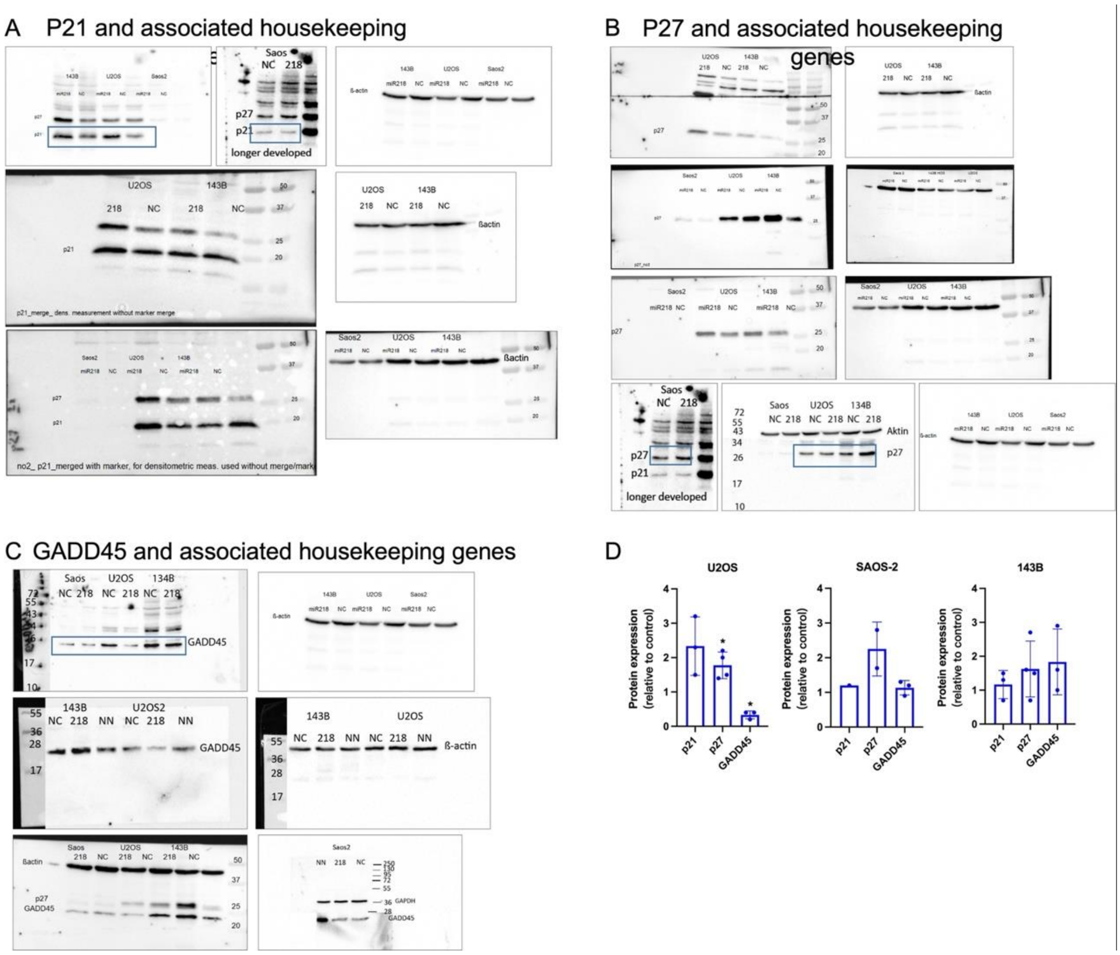

Supplementary Figure 4. Western blot images of P21 (A), P27 (B) GADD45 (C) and their relative density (D) in U2OS, 143B and SAOS-2 cells transfected with val-miR218 or NC miRNA. The experiments were conducted at least 3 times. The blots in blue frame are also present in Figure 5.

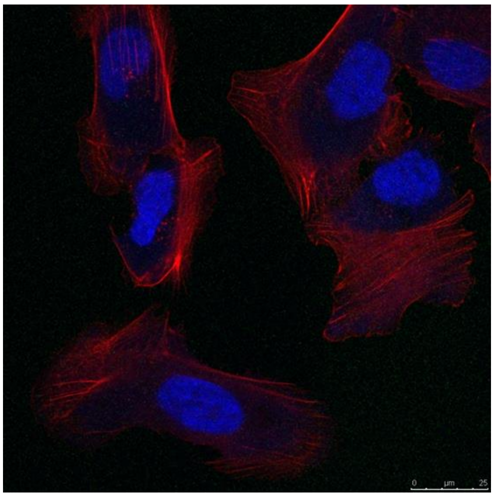

Supplementary Figure 5. Naked FAM-labeled val-miR218 was incubated with U2OS cells for 24 h. The uptake of FAM-labeled val-miR218 by U2OS was checked under a confocal microscope. The cell nucleus was stained by Hoechst 33342, and the cell skeleton was stained by phalloidin.
